# The Simultaneous Use of Bladder Epicheck^®^ and Urinary Cytology Can Improve the Sensitivity and Specificity of Diagnostic Follow-Up of Urothelial Lesions: Up-to-Date Data from a Multi-Institutional Cohort

**DOI:** 10.3390/diseases12090219

**Published:** 2024-09-18

**Authors:** Ludovica Pepe, Vincenzo Fiorentino, Cristina Pizzimenti, Giuseppe Riganati, Mariausilia Franchina, Marina Micali, Fernanda Russotto, Antonio Ieni, Giovanni Tuccari, Guido Fadda, Francesco Pierconti, Maurizio Martini

**Affiliations:** 1 Department of Human Pathology in Adult and Developmental Age “Gaetano Barresi”, University of Messina, 98125 Messina, Italy; ludopepe97@gmail.com (L.P.); vincenzo.fiorentino@unime.it (V.F.); cristinapizzimenti86@gmail.com (C.P.); giuseppe.riganati@studenti.unime.it (G.R.); mariausilia.franchina@studenti.unime.it (M.F.); micalimarina@yahoo.it (M.M.); russottofernanda@gmail.com (F.R.); antonio.ieni@unime.it (A.I.); gtuccari@unime.it (G.T.); guido.fadda@unime.it (G.F.); 2 Department of Life Sciences and Public Health, Catholic University of the Sacred Heart, Agostino Gemelli IRCCS University Hospital Foundation, 00168 Rome, Italy; francesco.pierconti@unicatt.it

**Keywords:** bladder cancer, Bladder Epicheck^®^, urinary cytology

## Abstract

**Background/Objectives:** Bladder cancer is a prevalent urinary system malignancy and urinary cytology is widely used for its screening and follow-up. A novel diagnostic tool called Bladder Epicheck^®^ (BE) is increasingly being used for monitoring the recurrence of non-muscle-invasive bladder cancer (NMIBC). The simultaneous use of BE and urinary cytology can increase the diagnostic performances in the follow-up of bladder neoplasms. **Methods:** In this multicenter study, we retrospectively evaluated the data of 322 patients in follow-up for a high-grade bladder carcinoma over a six-year period (from January 2018 to March 2024). The diagnostic performances of both cytology and BE and their combination were calculated using histology as gold standard. **Results:** Recurrences were diagnosed as high-grade urothelial carcinoma NMIBC in 18 cases, low-grade papillary NMIBC in 8 cases, and carcinoma in situ (CIS) in 4 cases. Cytological analysis correctly identified 26 out of 30 carcinomas, while 286 were correctly diagnosed as negative results. BE correctly identified 25 out of 30 carcinomas, 285 were correctly diagnosed as negative results. The combination of BE and urinary cytology correctly identified 29 out of 30 carcinomas, while 289 were correctly diagnosed as negative results. **Conclusions:** The combination of BE and cytology could be the most effective approach for follow-up diagnosis in patients with high-grade NMIBC, reducing unnecessary invasive procedures.

## 1. Introduction

Bladder cancer (BCa) is the most frequent malignancy of the urinary system and one of the most common cancers globally [1]. In Europe and the United States, bladder cancer accounts for 5% to 10% of all male cancers [2].

Bladder cancer and upper urothelial tract carcinoma are frequent illnesses with a high risk of recurrence, demanding ongoing care after first therapy [3]. Non-muscle-invasive bladder cancer (NMIBC) following transurethral resection is managed with monitoring, intravesical treatment, cystoscopy, and urine cytology. Urinary cytology is one of the most widely used screening and follow-up tests and shows a high sensitivity for high-grade lesions and carcinoma in situ (CIS), while it is less effective in identifying low-grade lesions [4]. In recent years, other follow-up techniques in urothelial carcinoma have been established; these techniques support and consolidate urinary cytology such as Bladder Epicheck^®^ (BE), which analyzes and identifies changes in DNA methylation that are involved in the urothelial carcinoma pathogenesis. DNA methylation is a process that alters gene expression without changing the DNA sequence [5]. In general, in cancer pathogenesis, this process can involve both tumor suppressor genes and oncogenes, which exhibit low or high expression when their promoters are hypermethylated or hypomethylated, respectively. BE is a potential non-invasive method for monitoring bladder cancer recurrence in people with a history of non-muscle-invasive bladder cancer (NMIBC). The BE test (Nucleix Ltd., Pekeris 3, Rehovot 7670203, Israel) is a non-invasive analysis that determines the methylation status of 15 genes, frequently involved in the urothelial cancer pathogenesis, to determine the existence of neoplastic cells in urine samples. The EpiScore, which ranges from 0 to 100, indicates a positive result (high risk for HGUC) when it is ≥60, whereas a score < 60 indicates a high probability of no bladder cancer or that the cancer is still in remission (negative or low risk for HGUC) [6]. An EpiScore of ≥90 is highly suggestive of HGUC.

BE has various benefits over standard urine biomarkers for identifying bladder cancer; in particular, BE has shown better sensitivity and negative predictive value in clinical investigations than recognized urinary biomarkers such as NMP22, BTA, UroVysion, and ImmunoCyt [7]. The BE test shows excellent sensitivity, particularly for high-grade tumors, which makes it an important tool for early detection and monitoring of bladder cancer [8]. Furthermore, BE has shown excellent specificity in identifying bladder cancer, which is critical for correct diagnosis and minimizing false positives [9]. Nowadays, many authors state that the simultaneous use of BE and urinary cytology can greatly increase the sensitivity and the specificity of diagnostic screening of urothelial lesions [10,11,12]. In fact, the combination of these two techniques may minimize needless investigations, potentially resulting in cost savings and reduced patient burden [13] and, in the future, could become an advantageous alternative to cystoscopy, considering some of its advantages, such as the non-invasive nature of the procedure and the reduction in costs compared to other techniques [14,15,16].

In this study, we report the results of the simultaneous use of BE and urinary cytology for the follow-up for bladder cancer from a large multi-institutional cohort, describing how this combination can improve the sensitivity and specificity of diagnostic follow-up of urothelial lesions.

## 2. Materials and Methods

We retrospectively evaluated the data of 322 patients in follow-up for an HG-NMIBC collected at AOU Policlinico G. Martino (Messina, Italy) and Fondazione Policlinico Universitario A.Gemelli IRCCS (Rome, Italy) in a period of time between January 2018 and March 2024.

The intravesical treatment was made with Bacillus Calmette Guerin (BCG) in 225 patients and Mitomycin C in 97 patients (Table 1). All patients had voided and washed urine samples collected (for cytology and BE) and had systematic random bladder biopsies with extra target biopsies for any suspicious lesion during cystoscopy for histology. According to the EAU guidelines, all the data we analyzed referred to the first follow-up examination after the end of intravesical therapy. The specimens were evaluated according to the 2017 tumor, node, and metastasis (TNM) classification and graded using both the 1973 and the 2004 World Health Organization (WHO) classifications [17] by two expert cytopathologists (FP and MM) with long experience (>15 years) in uropathology, and in cases where a consensus among the pathologists could not be reached, another expert uropathologist (GF) was consulted.

A patient was defined negative when cytology, cystoscopy, and histology were all negative. The primary endpoint of this study was to evaluate the sensitivity, specificity, negative and positive predictive value of cytology alone, BE alone, and a combination of both techniques.

### 2.1. Cytology

The urine samples were centrifuged for 10 min at 2000 g at room temperature. The resultant pellets were resuspended in Thin Prep PreservCyt solution and processed on the TP 5000 System (Hologic Inc., Marlborough, MA, USA). The cytological specimens were evaluated using the Papanicolau staining procedure, and the diagnosis was made using the Paris System for Reporting Urinary Cytology, which classified the cytological specimens as negative for high-grade urothelial carcinoma (NHGUC), atypical urothelial cells (AUCs), suspicious for high-grade urothelial carcinoma (SHGUC), or positive for high-grade urothelial carcinoma (HGUC) [18].

For statistical analysis, NHGUC was regarded negative, but AUCs, SHGUC, and HGUC were deemed positive.

### 2.2. Histology

All histology specimens obtained following cystoscopy were preserved with 10% buffered formalin for 12 to 48 h at room temperature. After obtaining paraffin-embedded tissue blocks, 4–5 µm thick slices were cut and stained with hematoxylin and eosin (H&E).

### 2.3. Bladder Epicheck^®^ Test

For the Bladder Epicheck^®^ test (Nucleix Ltd., San Diego, CA, USA), the urine samples were centrifuged twice at 1000 g for 10 min at room temperature. DNA, after extraction using a Bladder Epicheck^®^ DNA extraction kit, was digested using a methylathion-sensitive restriction enzyme, which cleaves DNA at its recognition sequence if it is unmethylated, following the manufacturer’s protocol. The samples were then processed for the PCR assay using the Bladder Epicheck^®^ test kit, and the findings were analyzed using Bladder Epi-Check software (Version 1.9). An EpiScore (a number between 0 and 100) was computed for samples that passed internal control validation. A score > or equal to 60 indicates a positive outcome, while a score <60 indicates a negative result. BE was conducted concurrently with urine cytology in voided specimens from the same patient.

### 2.4. Statistical Analysis

The sensitivity, specificity, odds ratio (OR), confidence interval (CI), negative predictive value (NPV), and positive predictive value (PPV) of cytology, BE, and their combination were calculated using histology as gold standard. Statistical analysis was carried out using GraphPad-Prism 5 software (Version 8.0) (GraphPad Software, San Diego, CA, USA) and MedCalc version 10.2.0.0 (MedCalc Software, Mariakerke, Belgium). Categorical variables were compared using the chi-square statistic and Fisher’s exact test. The region below the receiver operating characteristic (ROC) curve was determined and assessed for significance using the z test. A *p*-value < 0.05 was considered statistically significant.

### 2.5. Ethics

Ethical review and approval were waived for this study due to its retrospective nature. All patient data were collected anonymously, and written informed consent, as part of the routine diagnosis and treatment procedures, was obtained from patients or their guardians in accordance with Good Clinical Practice guidelines and the Declaration of Helsinki (1975, revised in 2013). The clinical information was retrieved from the patients’ medical records and pathology reports. The patients’ initials or other personal identifiers do not appear in any image.

## 3. Results

The mean age of the patients was 64 years (range 45–83 years). A total of 221 out of 322 patients were male (68.6%). All patients had an initial diagnosis of HG papillary NMIBC: 247 with T1 HG and 75 had CIS (Table 1).

The recurrences were diagnosed as high-grade urothelial carcinoma NMIBC in 18 cases, low-grade papillary NMIBC in 8 cases, and CIS in 4 cases. In particular, in the high-grade carcinoma group, 10 patients were diagnosed with HG papillary carcinoma Ta HG, 5 patients with HG papillary carcinoma T1 HG, 2 patients with HG papillary urothelial carcinoma with divergent differentiation (squamous differentiation), and 1 patient with HG papillary urothelial carcinoma with divergent differentiation (squamous and glandular aspects). All recurrent cases with a low-grade papillary NMIBC were Ta LG.

**Figure 1:**
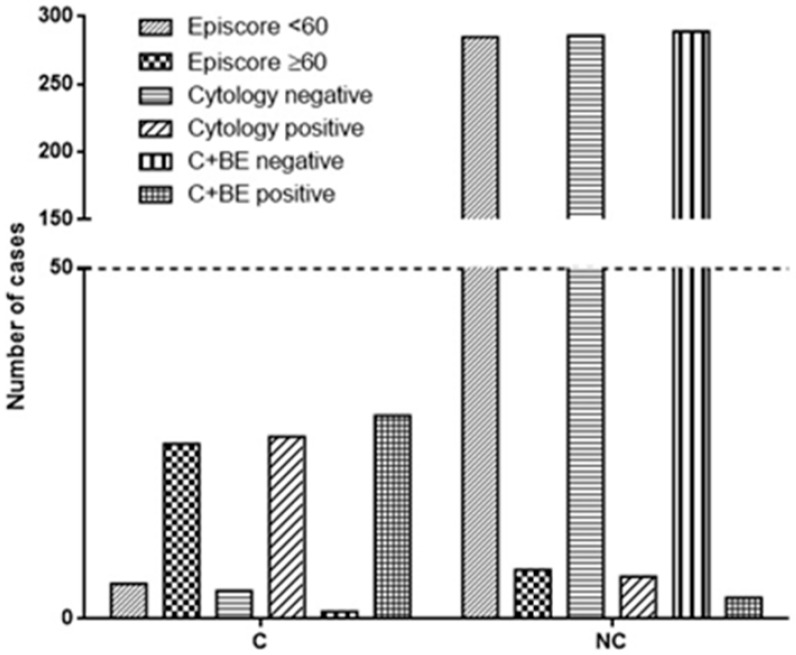
The figure illustrates the diagnostic accuracy of Bladder Epicheck^®^ (BE), cytology, and their combination. BE correctly identified 25 out of 30 carcinomas, while cytology accurately detected 26 out of 30. The combination of both techniques demonstrated the highest accuracy, correctly identifying 29 out of 30 carcinomas. In terms of negative results, BE correctly identified 285 out of 292 cases, cytology identified 286 out of 292 cases, and the combined approach identified 289 out of 292 cases (C + BE = cytology and Bladder Epicheck^®^; C = cancer at recurrence; NC = no cancer at recurrence).

Cytological analysis correctly identified 26 out of 30 carcinomas, while 4 cases of carcinomas were missed (Table 2, Figure 1). Moreover, 6 cases were erroneously identified as carcinoma, while 286 were correctly diagnosed as negative results (*p* < 0.0001, OR 309.8; 95% CI from 82.14 to 1169, Fisher’s exact test; Figure 1).

On the other side, BE correctly identified 25 out of 30 carcinomas, while 5 cases of carcinomas were missed. Of note, 7 cases were erroneously identified as carcinoma, while 285 were correctly diagnosed as negative results (*p* < 0.0001, OR 203.6; 95% CI from 60.19 to 688.6, Fisher’s exact test; Figure 1). Globally, the cytological analysis showed a sensitivity of 86.67% (range from 69.28% to 96.24%), specificity of 97.95% (95.12% to 99.03%), PPV of 81.25% (65.97% to 90.64%), and negative predictive value (NPV) of 98.62% (96.63% to 99.44%; Table 3). The same evaluation performed for BE resulted in 83.33% (65.28% to 94.36%) sensitivity, 97.60% (95.12% to 99.03%) specificity, 78.13% (62.80% to 88.31%) PPV, and 98.28% (96.24% to 99.22%; Table 3) NPV.

**Figure 2:**
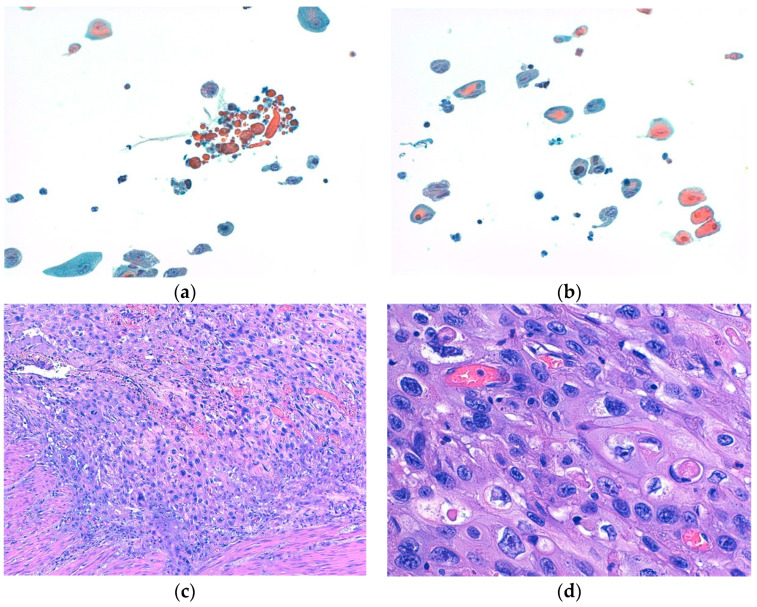
(**a**,**b**) show representative cytological aspects of neoplastic urothelial cells with divergent differentiation (squamous differentiation), with nuclear atypia and prominent nucleoli ((**a**,**b**): Papanicolaou stain, ×200). (**c**,**d**) show the corresponding histological features of high-grade urothelial carcinoma with divergent differentiation (squamous differentiation), made up of pleomorphic cells with marked nuclear atypia, prominent nucleoli, and an abundance of eosinophilic cytoplasms ((**c**): hematoxylin and eosin (H&E) stain, ×100; (**d**): H&E stain, ×400).

Moreover, both BE and cytology yielded false-negative results (5 and 4, respectively). Specifically, BE failed to detect five different histotypes of bladder HGUCs, namely, two CIS and three cancers with divergent differentiation, including two with squamous differentiation and one with both glandular and squamous differentiation (Figure 2). Cytology, on the other hand, missed three low-grade bladder carcinomas and one CIS.

The combination of the two techniques (BE + urinary cytology) correctly identified 29 out of 30 carcinomas, while 1 case (histologically diagnosed as CIS) was missed. Moreover, 3 cases were erroneously identified as carcinoma, while 289 were correctly diagnosed as negative results (*p* < 0.0001, OR 2794; 95% CI from 281.3 to 27,745, Fisher’s exact test). The two techniques together showed a sensitivity of 96.67% (82.78% to 99.92%), specificity of 98.97% (97.03% to 99.79%), PPV of 90.62% (75.79% to 96.76%), and negative predictive value (NPV) of 99.66% (97.68% to 99.95%; Table 3).

We also analyzed the diagnostic performance of BE, cytology, and their combination in detecting each type of carcinoma enclosed in our casuistry (Table 4, Table 5 and Table 6). Overall, the combination of cytology and BE showed the highest sensitivity for detecting all three types of carcinoma. The specificity of all the diagnostic modalities was consistently high, indicating a low rate of false-positive results. PPV was also notably high for the combined approach, while NPV was exceptionally high for all modalities, particularly for the combined approach, indicating high reliability in ruling out the presence of carcinoma.

## 4. Discussion

In our study, 30 out of 322 patients (9.3%) experienced a relapse. Of these, four were not detected by cytology, including three low-grade carcinomas (16.6%) and one CIS (8.3%). Additionally, BE failed to identify five carcinomas, all of which were high-grade (41.6%). These included two CIS, two with divergent differentiation (specifically, squamous features), and one with both glandular and squamous features.

As evident from the results, the cytological analysis failed to identify four low-grade cases. This is consistent with literature data indicating that the sensitivity and specificity of cytology vary depending on the grade of bladder cancer. The sensitivity increases for high-grade carcinomas and decreases for low-grade carcinomas. In fact, the sensitivity of cytology for high-grade carcinomas and CIS can range between 80% and 90% [19].

The Paris System for Reporting Urinary Cytology (TPS) is a well-established model for urinary diagnostics. However, it is not immune to occasional false positives [20]. Factors such as reactive atypia and cellular degeneration of urothelial cells can influence these results [21].

BE did not identify five high-grade carcinomas: two CIS, highlighting the same limitations as cytology, and three other high-grade carcinomas with divergent differentiation (glandular and squamous aspects). BE is quite a simple test based on only 15 DNA methylation biomarkers [6] and several factors, such as molecular mechanisms, different bladder cancer types, urine collection methods, and DNA processing, and can lead to false negatives. Identifying these factors could improve BE accuracy. In our casuistry, the presence of false negatives was probably related to molecular processes beyond methylation that might contribute to bladder cancer development, hindering its detection by BE. Alternatively, we could hypothesize that divergent differentiation might be associated with the epigenetic silencing of genes other than those analyzed by BE. Also, Fangdie Y. et al. explored the association between DNA methylation patterns and molecular subtypes, suggesting DNA methylation’s role in shaping bladder tumor molecular characteristics and potential use in predicting tumor behavior and therapy response [22].

Overall, methylation is a complex and dynamic phenomenon in bladder carcinoma, and DNA methylation patterns are very heterogeneous in such a pathology. Studies have shown that the methylation status of specific genes can vary significantly between different tumor stages and grades, thus influencing the outcomes of urinary methylation analyses [23]. Interestingly, Kim et al. reported that the differentiation of bladder tumors into less aggressive luminal subtypes, based on Hedgehog signaling activity, can impact growth rate and potentially influence the detection sensitivity of methylation analysis, leading to variations in false-negative rates [24]. Therefore, discovering and combining biomarkers beyond DNA methylation indicators could provide a more comprehensive understanding of bladder cancer and enhance test sensitivity [20]. However, our false-negative cohort is too limited for drawing conclusions regarding the impact of divergent differentiation on the diagnostic capacity of BE. Therefore, we need further research with larger cohorts to explore this aspect in more detail.

Nevertheless, in our study, the combined use of cytology and BE proved to be very effective, failing to identify only one carcinoma, histologically diagnosed as CIS. Notably, the only CIS missed by cytology was detected by BE [25]. Also, only three cases were erroneously classified as carcinoma by both cytology and BE. It is plausible that these false-positive diagnoses may be attributed to intravesical therapy, which can induce cellular histological alterations and potentially impact DNA methylation status [26,27].

Overall, our findings suggest that the combined use of BE and cytology in bladder cancer can enhance diagnostic accuracy, significantly reducing both false-negative and false-positive cases. This consideration is also confirmed when considering the diagnostic performance of the three different diagnostic modalities in detecting each type of carcinoma enclosed in our casuistry. In fact, the combination of cytology and BE emerged as the most effective approach, offering the highest sensitivity and PPV across all three types of carcinoma. However, the choice between BE alone and cytology alone might depend on the specific clinical context and the clinical suspicion. In fact, if a low-grade papillary NMIBC is the primary concern, BE alone might be sufficient due to its perfect sensitivity for this type. On the other hand, if high-grade NMIBC is suspected, cytology alone could be considered due to its perfect sensitivity and NPV for this type. In any case, the high specificity and NPV of all three modalities underscore their value in ruling out carcinoma in patients with negative results.

Urinary cytology, the standard technique for bladder cancer follow-up, offers advantages such as low cost and high sensitivity for high-grade tumors. However, it also presents disadvantages, including operator dependence, requesting substantial expertise, and potential patient discomfort during urine collection.

Conversely, BE has demonstrated high sensitivity and specificity in detecting bladder cancer, although it may be less effective for certain histological variants. BE offers advantages such as being operator-independent and not causing patient discomfort during urine collection. However, its significantly higher cost compared to cytology limits its widespread adoption [27].

Combining BE and cytology for bladder cancer diagnosis could be effective in monitoring recurrence and potentially reducing the need for invasive cystoscopies in patients with suspected NMIBC [28,29,30]. This approach could help minimize unnecessary cystoscopies, leading to improved patient care, a reduced risk of infections, and decreased healthcare costs, which are significantly higher than the costs associated with the combined use of cytology and BE analysis.

Lastly, the improved sensitivity of the combined approach may enable earlier detection of tumor recurrence, allowing for prompt intervention and potentially improving treatment outcomes. However, further research is needed in larger and different cohorts to confirm our results.

## 5. Conclusions

Our study analyzes the use of two follow-up techniques in bladder cancer diagnosis: urinary cytology and BE. Urinary cytology is the standard for follow-up of bladder cancer and has advantages such as low cost and high sensitivity for high-grade tumors. However, BE demonstrated high sensitivity and specificity in detecting bladder cancer but not in certain subtypes of high-grade carcinomas. The combination of BE and cytology could be the most effective approach for follow-up diagnosis in patients with history of HG-NMIBC.

We suggest that combining both methods could be the most effective approach for initial diagnosis in patients with suspected bladder cancer, leveraging the high sensitivity of BE for low-grade urothelial carcinomas and the high sensitivity of cytology for high-grade carcinomas; it could be a valuable tool for monitoring patients with NMIBC for recurrence, potentially reducing the need for invasive cystoscopies.

The results could contribute to the development of new risk stratification tools for bladder cancer, aiding in the identification of high-risk patients who may require more aggressive treatment. Combining BE and cytology can improve diagnostic accuracy and potentially reduce the need for invasive procedures, ultimately leading to better patient care and outcomes and also a reduction in public health costs.

## Figures and Tables

**Table 1:** Clinical and pathological characteristics of the study population.

**Age**	64 (range from 48 to 83)
**Sex**	Male = 221; Female = 101
**First diagnosis of HG papillary NMIBC ^1^**	132 T1 HG 75 CIS ^2^
**Intravesical treatment**	^3^ BCG in 225 patients; Mitomycin C in 97 patients
**Diagnosis at recurrence**	18 cases of HG ^4^ urothelial carcinoma NMIBC 8 cases of LG ^5^ papillary NMIBC 4 cases CIS

^1^ NMIBC = non-muscle-invasive bladder carcinoma; ^2^ CIS = carcinoma in situ; ^3^ BCG = Bacillus Calmette Guerin; ^4^ HG = high grade; ^5^ LG = low grade.

**Table 2:** Diagnostic accuracy of cytology, BE, and their combination in the detection of urothelial lesions, as determined via comparisons with histological findings.

	Positive Histology	Negative Histology
**Positive cytology**	26	6
**Negative cytology**	4	286
**Positive BE ^1^**	25	7
**Negative BE**	5	285
**Positive BE + cytology**	29	3
**Negative BE + cytology**	1	289

^1^ BE= Bladder Epicheck^®^.

**Table 3:** Comparison of performance between cytology, BE, and the two techniques together.

	Cytology	BE	Cytology + BE
**Sensitivity**	86.67% (95% CI ^1^ 69.28% to 96.24%)	83.33% (95% CI 65.28% to 94.36%)	96.67% (95% CI 82.78% to 99.92%)
**Specificity**	97.95% (95% CI 95.12% to 99.03%)	97.60% (95% CI 95.12% to 99.03%)	98.97% (95% CI 97.03% to 99.79%)
**PPV**	81.25% (95% CI 65.97% to 90.64%)	78.13% (95% CI 62.80% to 88.31%)	90.62% (95% CI 75.79% to 96.76%)
**NPV**	98.62% (95% CI 96.63% to 99.44%)	98.28% (95% CI 96.24% to 99.22%)	99.66% (95% CI 97.68% to 99.95%)

^1^ CI, confidence interval.

**Table 4:** Diagnostic performance of BE for detecting different types of bladder carcinoma in our casuistry.

	CIS	High-Grade NMIBC	Low-Grade Papillary NMIBC
**Sensitivity**	50.00% (95% CI ^1^ 6.76% to 93.24%)	83.33% (95% CI 58.58% to 96.42%)	100.00% (95% CI 63.06% to 100.00%)
**Specificity**	100.00% (95% CI 98.74% to 100.00%)	98.63% (95% CI 96.53% to 99.63%)	98.97% (95% CI 97.03% to 99.79%)
**PPV**	100.00% (95% CI 15.81% to 100.00%)	78.95% (95% CI 58.10% to 91.03%)	72.73% (95% CI 46.38% to 89.15%)
**NPV**	99.32% (95% CI 98.21% to 99.74%)	98.97% (95% CI 97.16% to 99.63%)	100.00% (95% CI 98.73% to 100.00%)

^1^ CI, confidence interval.

**Table 5:** Diagnostic performance of cytology for detecting different types of bladder carcinoma in our casuistry.

	CIS	High-Grade NMIBC	Low-Grade Papillary NMIBC
**Sensitivity**	75.00% (95% CI ^1^ 19.41% to 99.37%)	100.00% (95% CI 81.47% to 100.00%)	62.50% (95% CI 24.49% to 91.48%)
**Specificity**	99.32% (95% CI 97.55% to 99.92%)	99.32% (95% CI 97.55% to 99.92%)	99.32% (95% CI 97.55% to 99.92%)
**PPV**	60.00% (95% CI 25.22% to 86.97%)	90.00% (95% CI 69.34% to 97.28%)	71.43% (95% CI 36.23% to 91.67%)
**NPV**	99.66% (95% CI 98.15% to 99.94%)	100.00% (95% CI 98.74% to 100.00%)	98.98% (95% CI 97.53% to 99.58%)

^1^ CI, confidence interval.

**Table 6:** Diagnostic performance of cytology + BE for detecting different types of bladder carcinoma in our casuistry.

	CIS	High-Grade NMIBC	Low-Grade Papillary NMIBC
**Sensitivity**	75.00% (95% CI ^1^ 19.41% to 99.37%)	100.00% (95% CI 81.47% to 100.00%)	100.00% (95% CI 63.06% to 100.00%)
**Specificity**	99.66% (95% CI 98.11% to 99.99%)	99.66% (95% CI 98.11% to 99.99%)	99.66% (95% CI 98.11% to 99.99%)
**PPV**	75.00% (95% CI 28.13% to 95.83%)	94.74% (95% CI 71.78% to 99.22%)	88.89% (95% CI 53.07% to 98.26%)
**NPV**	99.66% (95% CI 98.16% to 99.94%)	100.00% (95% CI 98.74% to 100.00%)	100.00% (95% CI 98.74% to 100.00%)

^1^ CI, confidence interval.

## Data Availability

The original contributions presented in this study are included in this article, further inquiries can be directed to the corresponding author.
